# Chronic Senolytic Treatment and/or Aerobic Exercise Reduce Senescence and Improve Myocardial Function During Aging in the Heart

**DOI:** 10.1111/acel.70759

**Published:** 2026-10-07

**Authors:** Ryan T. Bevington, Amanda L. Johnson, Briana L. Hockey, Val A. Fajardo, Gianni Parise

**Affiliations:** ^1^ Department of Kinesiology McMaster University Hamilton Ontario Canada; ^2^ Department of Kinesiology Brock University St. Catherines Ontario Canada

**Keywords:** aging, exercise, heart, myocardium, senescence, senolytics

## Abstract

Aging is the predominant risk factor for cardiac dysfunction, driven primarily through a decline in cardiomyocyte and overall myocardial performance. Cellular senescence is becoming increasingly linked to its development, as senescent cardiomyocytes and interstitial cells accumulate with age and exhibit persistent DNA damage, mitochondrial dysfunction, and inflammation that collectively impair ventricular relaxation and contractility. Senolytics, which selectively eliminate senescent cells, effectively clear cardiac senescence and its burden, while aerobic exercise has shown promise as a non‐pharmacological intervention capable of modulating senescence. Despite this, neither approach has been evaluated as a long‐term preventive strategy or whether combining exercise with senolytics produces an additive effect. We hypothesized that when combined, chronic senolytics and aerobic exercise would produce a synergistic clearance of senescent myocardial cells leading to improved cardiac function. Male and female C57BL/6N mice (12‐months‐old) were randomly allocated to each group and received their intervention for 9‐months: naturally aged (VEH, *n* = 12), aerobic exercise (EX, *n* = 11), senolytics Dasatinib + Quercetin (D + Q) (SEN, *n* = 15) or both combined (SENEX, *n* = 16). Chronic D + Q, exercise, or both, significantly lowered the proportion of p16+, p21+, and γ‐H2AX+ cardiomyocytes and interstitial cells, while improving myocardial performance and diastolic function compared to naturally aged mice. At the whole‐heart level, protein expression of p16 and p21 increased with age, but was not altered alongside SASP by any intervention. Collectively, these findings demonstrate that long‐term exercise or senolytics limit senescence at the cellular level within the myocardium, and improve ventricular function, positioning exercise or senolytics as promising strategies to mitigate age‐related cardiac dysfunction.

## Graphical abstract

Middle aged mice (12‐months‐old) administered senolytics and/or aerobic exercise for 9 months showed a significantly lower accumulation of senescent cardiomyocytes and improved myocardial function compared to mice aged naturally, independent of changes to the SASP.

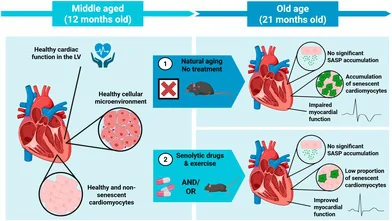

## 1 Introduction

Aging of the heart is accompanied by marked structural, molecular, and functional changes within its muscle component, the myocardium. These alterations manifest at the cellular level and progressively translate into functional deficits, increasing the risk of adverse outcomes like heart failure (Ribeiro et al. 2023). In the context of a rapidly aging population, this poses a growing threat to cardiac health, healthcare systems, and overall well‐being.

A likely contributor is cellular senescence. First described by Hayflick and Moorhead (1961), senescence was characterized as an irreversible cell cycle arrest but has now evolved into a complex cellular state that includes mitochondrial dysfunction, oxidative stress, DNA damage, and a senescence associated secretory phenotype (SASP) (Hernandez‐Segura et al. 2018). Senescent cells can arise from replicative exhaustion or in response to molecular and environmental stressors. These stressful stimuli activate pathways like the DNA damage response (DDR), which propagate a permanent exit from the cell cycle via the upregulation of cyclin dependent kinase (CDK) inhibitors, such as canonical senescence markers p16 and p21 (Herranz and Gil 2018; Robles and Adami 1998). Furthermore, senescent cells upregulate pro‐survival networks called senescent cell anti‐apoptotic pathways (SCAPs), enabling apoptotic resistance (Wang 1995; Zhu et al. 2015). A growing body of evidence links their excessive accumulation with age to declines in cardiac health and function (Chen et al. 2022; Luan et al. 2024).

This is thought to be driven largely through the SASP, which contains pro‐inflammatory, pro‐fibrotic, metabolic, and matrix remodeling components (Coppé et al. 2010). While SASP remains a complex heterogenous signature, conserved components like interleukin‐6 (IL‐6), tumor necrosis factor alpha (TNF‐α), nuclear factor kappa‐B (NF‐κB), and transforming growth factor beta (TGF‐β) drive increases in inflammation, fibrosis, reactive oxygen species (ROS), and oxidative stress throughout the cardiac environment (Khavinson et al. 2023). This results in disrupted intercellular communication, mitophagy, autophagy and senescent cell accumulation through the paracrine induction of senescence (Hoare et al. 2016).

In the aged myocardium, there are pronounced increases in senescent myocardial cells, namely cardiomyocytes (CMs), fibroblasts, and endothelial cells (Chimenti et al. 2003). This elevated presence of senescent cells has been linked to heightened inflammation, mitochondrial dysfunction, and maladaptive cardiac remodeling (Luan et al. 2024; Martini et al. 2021). Moreover, senescent CMs also display impaired shortening, re‐lengthening, and calcium handling (Lim et al. 2000). As a result, these alterations increase myocardial stiffness and can impair the contractility and relaxation of the left ventricle (LV), yielding progressive declines in systolic and diastolic function. These observations position myocardial senescence as a central driver of age‐related cardiac dysfunction.

Therefore, strategies aimed at reducing cellular senescence and attenuating its burden are crucial for limiting the age‐associated functional decline in the heart. One such approach employs the pharmacological clearance of senescence cells using drugs termed senolytics. Senolytics selectively target SCAPs to induce apoptosis in senescent cells, with the most widely studied agents being a combination of dasatinib and quercetin (D + Q) or navitoclax (Zhu et al. 2015). Despite few investigations in cardiac tissue, previous work demonstrates that D + Q can reduce the gene expression of senescence marker p16 (Lewis‐McDougall et al. 2019) and improve ejection fraction (EF) (Zhu et al. 2015), while navitoclax treatment can significantly reduce the proportion of senescent CMs, fibroblasts, and improve measures of systolic and diastolic function (Anderson et al. 2019; Jia et al. 2020; Walaszczyk et al. 2019). Therefore, senolytics show promise as an effective strategy to attenuate senescence and its burden.

An alternative, less invasive, and more cost‐effective strategy is aerobic exercise. In addition to its well‐established benefits for cardiac and mitochondrial health, there is growing interest in its potential to attenuate cellular senescence (Qiu et al. 2025). In fact, recent work suggests that aerobic exercise imparts a “natural” senolytic‐like effect; however, this has been examined predominantly in skeletal muscle (Jean et al. 2024; Zhang et al. 2022). To date, only one study has assessed this in the heart, reporting that 8 weeks of voluntary wheel running reduced myocardial expression of the senescence markers p16 and p53, although this was observed in young mice (Werner et al. 2008).

Collectively, both senolytics and exercise have emerged as promising strategies to alleviate cellular senescence and improve cardiac function during aging, but no work has investigated combining senolytics and exercise together. Moreover, the chronic effect of senolytics on cardiac tissue remains poorly defined, particularly with D + Q, as the longest study lasted 2 months in aged mice (Lewis‐McDougall et al. 2019). This leaves unresolved knowledge gaps regarding the synergy between senolytic and exercise interventions, and whether prolonged senolytic administration can prevent the age‐associated accumulation of senescent cells in the myocardium.

Here, we aimed to address these gaps and treated adult mice (12‐months old) with 9‐months of a vehicle, senolytics (D + Q), aerobic exercise, or both combined until the beginning of old age (21‐months old). Our primary objective was to uncover the effects of chronic exercise and senolytics on the development of senescence within the myocardium and its resulting influence on cardiac function. We hypothesized that when combined, senolytics and exercise would reduce senescence and its burden, leading to improved cardiac function compared to vehicle‐treated mice and either intervention alone.

## 2 Results

### 2.1 Anthropomorphic and General Cardiac Function Parameters Following the 9‐Month Intervention

Standard anthropomorphic and cardiac function parameters were used to assess overall health of the mice and heart. After 9‐months, body weight did not differ significantly between the intervention groups (*p* > 0.05) (Table 1), but the YOUNG mice had a significantly smaller body weight than all the groups (*p* < 0.05) (Table 1). Similarly, dissected hearts, perfused and weighed after collection from all the groups were significantly heavier than the YOUNG group (*p* < 0.05) (Table 1). Moreover, LV mass was markedly elevated in all groups compared the YOUNG mice (*p* < 0.05) (Table 1), with no observed differences across the intervention groups (*p* > 0.05) (Table 1). As the YOUNG mice were significantly smaller in size, we opted to normalize metrics of heart size to body weight, which revealed that relative heart weight and LV mass was not markedly different across all groups (*p* > 0.05) (Table 1). Heart rate was consistent across the groups, except the SENEX group had a higher heart rate than the YOUNG (*p* < 0.05) (Table 1).

**TABLE 1:** Anthropomorphic measurements.

Measure	YOUNG	VEH	EX	SEN	SENEX
Body weight (g)	24.44 ± 2.32	39.95 ± 7.71**	36.45 ± 8.01**	40.52 ± 8.52**	35.85 ± 4.50**
Heart weight; raw value (mg)	145.6 ± 18.18	218.9 ± 35.50**	188.6 ± 42.07**	209.1 ± 28.69**	200.5 ± 36.82**
Heart weight; normalized to BW (mg/g)	5.953 ± 0.4035	5.329 ± 0.7928	5.174 ± 1.571	5.117 ± 0.9270	5.412 ± 0.9074
Heart rate (bpm)	370.3 ± 29.73	393.6 ± 47.44	398.2 ± 24.40	400.6 ± 27.99	418.2 ± 32.40**
LV mass; raw value (mg)	125.9 ± 33.06	213.9 ± 50.32**	215.6 ± 50.62**	198.9 ± 30.90**	197.9 ± 48.15**
LV mass; normalized to body weight (mg/g)	5.130 ± 1.101	5.209 ± 1.146	5.847 ± 1.427	4.837 ± 0.8932	5.478 ± 1.463

*Note:* Basic physical characteristics of the body and heart after 9‐months of treatment. VEH‐SENEX: 21‐months old, YOUNG: 4‐months old. YOUNG (*n* = 10), VEH (*n* = 10), EX (*n* = 9), SEN (*n* = 13), and SENEX (*n* = 14). All data is presented as mean ± SD.

** Denotes significantly different from YOUNG at *p* < 0.05.

We also utilized high frequency ultrasound (HFU) to assess the general function of the heart and derived measures of cardiac output (CO), stroke volume (SV), end diastolic volume (EDV), and end systolic volume (ESV) (Table 2). We assessed LV filling metrics through the E (early filling) and A (atrial filling) wave velocities, acceleration, deceleration time (DT), and ejection time (ET) (Table 2), as well as a general proxy of diastolic function in the E/A ratio (Figure 1). Finally, the EF and fractional shortening (FS) were used to represent systolic function (Figure 1). From this, we report no differences for the following measures: ESV, E wave, A wave, acceleration, and DT (*p* > 0.05) (Table 2). However, the VEH mice had a significantly lower relative CO than the YOUNG mice (*p* < 0.05) (Table 2), which was not different from any of the interventions. Moreover, only the VEH and SEN mice had a markedly smaller relative SV than the YOUNG mice, which was also underpinned by a significantly smaller EDV in the SEN mice (*p* < 0.05) (Table 2). Finally, the YOUNG hearts' relative ET was prolonged compared to all the other groups (*p* < 0.05) (Table 2).

**TABLE 2:** General cardiac function and LV filling metrics largely unaffected by age or treatment.

Measure	YOUNG	VEH	EX	SEN	SENEX
Cardiac output (mL/min·g)	0.6321 ± 0.1286	0.4376 ± 0.08640**	0.5696 ± 0.09219	0.5087 ± 0.1537	0.5724 ± 0.1441
Stroke volume (μL/g)	1.716 ± 0.3564	1.129 ± 0.2431**	1.431 ± 0.2215	1.266 ± 0.3750**	1.464 ± 0.4008
End diastolic volume (μL/g)	3.435 ± 0.7956	2.718 ± 0.8736	2.853 ± 0.5746	2.408 ± 0.5721**	2.733 ± 0.5026
End systolic volume (μL/g)	1.719 ± 0.5029	1.588 ± 0.7701	1.422 ± 0.5245	1.142 ± 0.3516	1.268 ± 0.3662
E wave (mm/s)	436.6 ± 124.0	385.4 ± 80.74	402.9 ± 73.03	438.7 ± 54.39	372.6 ± 62.43
A Wave (mm/s)	158.9 ± 30.66	213.0 ± 94.62	133.0 ± 50.70	214.4 ± 83.04	146.8 ± 52.30
Acceleration (m/s^2^)	30.56 ± 8.439	29.41 ± 8.423	40.80 ± 21.92	39.05 ± 14.37	39.28 ± 14.42
Deceleration time (ms)	23.12 ± 9.164	17.50 ± 6.797	21.96 ± 4.709	17.52 ± 9.288	20.48 ± 10.86
Ejection time (ms/g)	3.230 ± 0.7506	1.381 ± 0.3966**	1.669 ± 0.5296**	1.559 ± 0.4597**	1.585 ± 0.4139**

*Note:* General proxies of cardiac function and LV filling dynamics after 9‐months of treatment. VEH‐SENEX: 21‐months old, YOUNG: 4‐months old. YOUNG (*n* = 10), VEH (*n* = 10), EX (*n* = 9), SEN (*n* = 13), and SENEX (*n* = 14). Metrics sensitive to mouse size were normalized to body weight: cardiac output, stroke volume, end diastolic volume, end systolic volume, and ejection time. All data is presented as mean ± SD.

** Denotes significantly different from YOUNG at *p* < 0.05.

**FIGURE 1:**
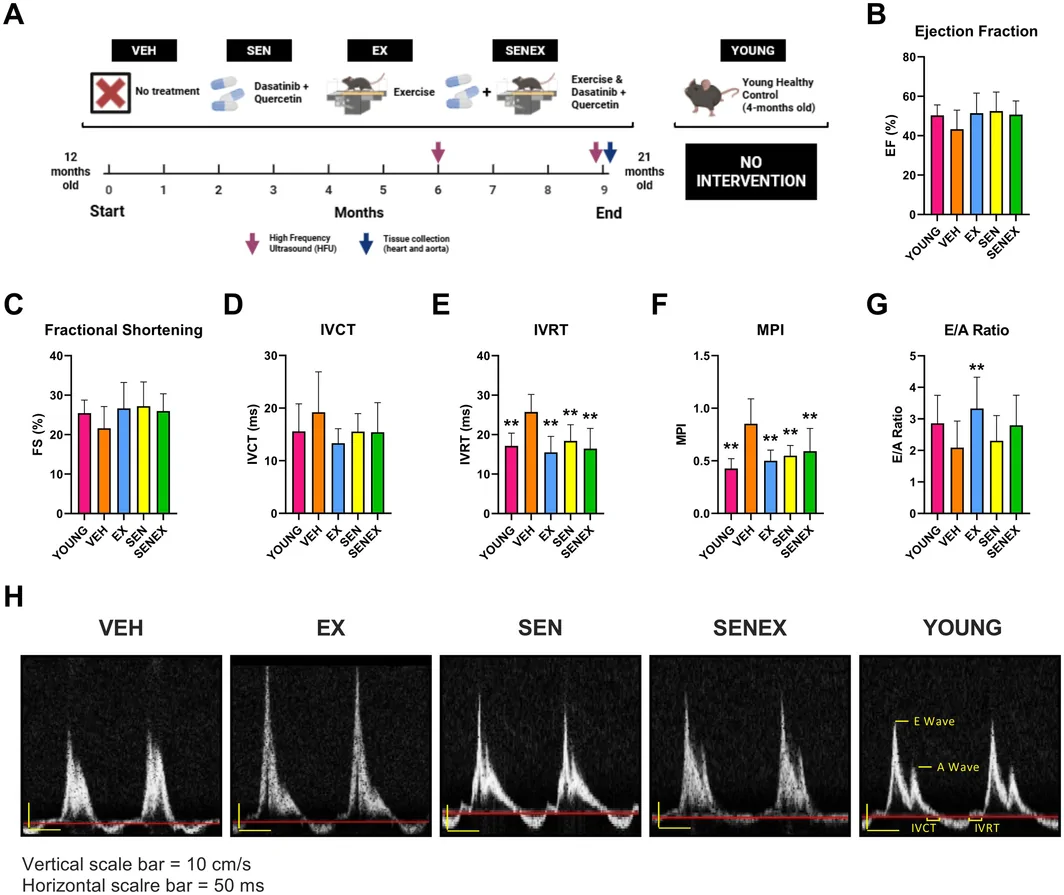
Mice given aerobic exercise, senolytics, or both combined for 9‐months display superior myocardial function to naturally aged mice. All treatment groups show better myocardial function compared to the naturally aged vehicle. (A) Experimental outline and groups. (B, C) Systolic function parameters, including the ejection fraction (EF) (B) and fractional shortening (FS) (C). (D–F) Myocardial function measurements, including the isovolumetric contraction time (IVCT) in ms (D), isovolumetric relaxation time (IVRT) in ms (E), and myocardial performance index (MPI) (F). (G) Diastolic function measure in the E/A ratio. (A‐G) YOUNG in pink (*n* = 10), VEH in orange (*n* = 10), EX in blue (*n* = 9), SEN in yellow (*n* = 13), and SENEX in green (*n* = 14). (H) Representative HFU scans in PW mode; yellow annotations denote specific measures. All data is presented as mean ± SD. ** denotes value significantly different from VEH at *p* < 0.05.

### 2.2 Exercise, Senolytics, or Both Combined Improve Myocardial Function

Finally, we assessed myocardial function of the LV, using the isovolumetric contraction time (IVCT) and isovolumetric relaxation time (IVRT), then used them to calculate the myocardial performance index (MPI) or Tei Index (MPI = [IVCT+IVRT]/ET). While there was no significant difference in IVCT across the groups (*p* > 0.05) (Figure 1D), the VEH mice displayed a significantly higher IVRT and MPI (25.7 ± 4.46 s and 0.85 ± 0.24) compared to EX (15.5 ± 4.03 s and 0.50 ± 0.10), SEN (18.4 ± 4.09 s and 0.55 ± 0.10), SENEX (16.4 ± 5.15 s and 0.59 ± 0.22), and YOUNG mice (17.1 ± 3.25 s and 0.43 ± 0.09) (*p* < 0.01) (Figure 1E,F). VEH hearts exhibited ~40% longer IVRT and ~44% higher MPI than the next highest intervention groups (IVRT: SEN; MPI: SENEX). Systolic function (EF and FS) trended lower in VEH but did not reach significance (*p* > 0.05) (Figure 1B–C). In contrast, EX mice showed a 1.60‐fold higher E/A ratio compared to VEH (3.33 ± 0.99 vs. 2.09 ± 0.84; *p* < 0.05) (Figure 1G), suggesting improved diastolic function.

### 2.3 Exercise, Senolytics, or Both Combined Reduce Markers of Senescence in Different Cardiac Cell Populations

To quantify senescent cells, we employed the immunofluorescence detection of canonical senescence markers p16, p21, and γ‐H2AX in CMs and interstitial cells (ICs) within the heart. The criteria used to identify positive cells are outlined in our methods and depicted in Figure 2A.

**FIGURE 2:**
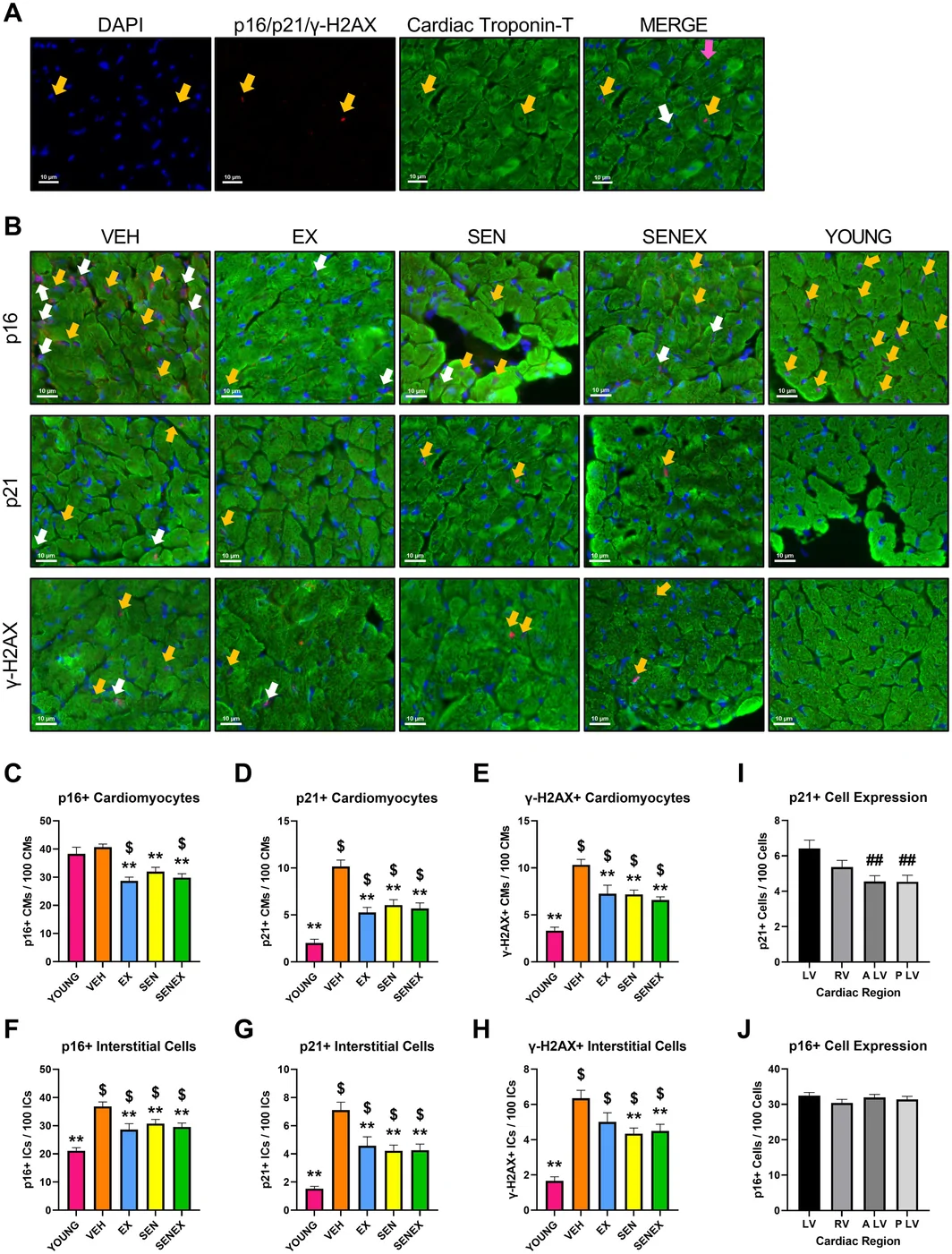
9‐months of senolytics, exercise, or both combined lower senescent marker expression in cardiac cells during aging. (A) Positive cell selection criteria. DAPI (blue), senescent marker (red), cardiac troponin‐T (green), and MERGE. White arrow denotes cardiomyocyte (CM), yellow arrow denotes positive cardiomyocyte, pink arrow denotes interstitial cell (IC). (B) Representative images for each group: VEH, EX, SEN, SENEX, and YOUNG (left→right), plus each marker: p16, p21, γ‐H2AX (top→down). Yellow arrows denoting a + senescent marker in the CM and white arrows denoting a + senescent marker in the IC. Quantitative analysis of (C) p16+ CMs (D) p21+ CMs (E) γ‐H2AX+ CMs (F) p16+ ICs (G) p21+ CMs (H) γ‐H2AX+ ICs. (C–H) YOUNG in pink (*n* = 10), VEH in orange (*n* = 12), EX in blue (*n* = 11), SEN in yellow (*n* = 15), and SENEX in green (*n* = 16). Values are expressed as positive cell occurrence per 100 cells counted. (I‐J) p16 and p21 cell expression across different areas of the heart: left ventricle (LV), right ventricle (RV), anterior LV (A LV), and posterior LV (P LV). All data is presented as mean ± SD. Scale bars: 10 μm. ** denotes value significantly different from VEH at *p* < 0.05. $ denotes value significantly different from YOUNG at *p* < 0.05. ## denotes value significantly less than the LV at *p* < 0.05.

After 9 months, EX (28.7 ± 4.4), SEN (32.0 ± 5.7), and SENEX (29.9 ± 5.3) hearts showed lower p16+ CMs compared to VEH (40.7 ± 3.8) (*p* < 0.05) (Figure 2C), with reductions of 29%, 21%, and 27%, respectively. In contrast, YOUNG mice (38.29 ± 7.5) did not differ from VEH or SEN (*p* > 0.05) (Figure 2C).

Similarily, EX (5.3 ± 1.7), SEN (6.1 ± 2.2), and SENEX (5.7 ± 2.3) groups had significantly lower p21+ CMs than the VEH group (10.2 ± 2.4) (*p* < 0.05) (Figure 2D). While YOUNG hearts had lower p21+ CMs (2.0 ± 1.3) than the VEH mice, and the rest of the interventions (*p* < 0.05) (Figure 2D). This was reflected in 48% less in the EX, 42% in the SEN, 44% in the SENEX, and 80% in the YOUNG compared to the VEH.

Using γ‐H2AX as a marker of DNA damage and senescence displayed similar patterns, with EX (7.3 ± 2.9), SEN (7.2 ± 1.8), and SENEX (6.6 ± 1.3) groups displaying fewer γ‐H2AX+ CMs than the VEH group (10.3 ± 2.0) (*p* < 0.05) (Figure 2E). Compared to the VEH, the EX and SEN hearts had 30% less γ‐H2AX+ CMs, while the SENEX had 36% less. YOUNG mice (3.3 ± 1.2) displayed lower γ‐H2AX+ CMs than the EX, SEN, and SENEX mice as well, including 68% less than the VEH (*p* < 0.05) (Figure 2E).

The same senescent markers were quantified in ICs between the CMs, the EX (28.6 ± 7.0), SEN (30.8 ± 5.4), and SENEX (29.6 ± 5.3) groups displayed fewer p16+ ICs than the VEH group (36.9 ± 5.5), showing 22%, 17%, and 20% reductions respectively (*p* < 0.05) (Figure 2F). Moreover, YOUNG mice had significantly less p16+ ICs than all other interventions (21.1 ± 3.4) (*p* < 0.05) (Figure 2F).

For p21, EX (4.6 ± 2.1), SEN (4.2 ± 1.5), and SENEX (4.3 ± 1.7) hearts had significantly fewer p21+ ICs than the VEH group (7.1 ± 2.0) (*p* < 0.05) (Figure 2G), with YOUNG hearts (1.5 ± 0.5) displaying less than all the groups (*p* < 0.05) (Figure 2G). This corresponded to 35% less p21+ ICs in the EX, 41% in the SEN, 39% in the SENEX, and 79% in the YOUNG hearts compared to the VEH.

For γ‐H2AX, SEN (4.3 ± 1.2) and SENEX (4.5 ± 1.5) had fewer γ‐H2AX+ ICs than the VEH mice (6.4 ± 1.6) (*p* < 0.05) (Figure 2H), but the EX (5.0 ± 1.7) showed no significant difference. However, YOUNG mice (1.7 ± 0.7) had markedly fewer γ‐H2AX+ interstitial cells than the intervention mice, with roughly 73% less than the VEH (*p* < 0.05) (Figure 2H).

Finally, we combined CM and interstitial cell counts to obtain senescent marker expression in total cells within the heart (Figure S2). These counts were completed in four separate regions of the heart: LV, RV, anterior LV (A LV), and posterior LV (P LV), which gave us a spatial representation of p21 and p16 expression in the myocardium (Figure 2I,J). There were 29% more p21+ cells in the center of the LV (6.4 ± 3.7) compared to the anterior and posterior edges (4.5 ± 2.6 and 4.5 ± 2.9) (*p* < 0.05) (Figure 2I), but no differences between the LV and RV (5.4 ± 2.9) and between the RV, A LV, and P LV (*p* > 0.05) (Figure 2I). There were no differences in p16+ cell expression across the heart (*p* > 0.05) (Figure 2J).

### 2.4 Naturally Aged Mice Demonstrate More Proxies of Senescence Than Young Healthy Mice, With No Mitigating Effect of Any Intervention at the Whole Heart Level

After we discovered a reduction in senescence markers within CM and IC populations, we wanted to investigate whether this would be reflected in protein and gene expression at the whole‐heart level. Accordingly, we performed RT‐qPCR and western blotting of canonical senescent markers from whole heart lysates.

Similar to our histological detection we found that the VEH mice had 3.07‐fold higher expression of p16 and 6.8‐fold higher p21 compared to the YOUNG (p16: 1.66 ± 1.08 vs. 0.54 ± 0.29, p21: 1.36 ± 1.16 vs. 0.20 ± 0.07; *p* < 0.05) (Figure 3A,B). The SEN hearts also had higher p21 (*p* < 0.05) (Figure 3B) but not p16 (Figure 3A), compared to the YOUNG mice (*p* < 0.05) (Figure 3B). There were no significant differences between the VEH and our intervention groups for both p16 and p21 expression despite blots appearing visually lower than the VEH (*p* > 0.05) (Figure 3A,B). We also analyzed p53 and senescence inducer phosphorylated p38MAPK (p‐p38MAPK) and found no differences in either expression across all the groups (*p* > 0.05) (Figure 3C,D).

**FIGURE 3:**
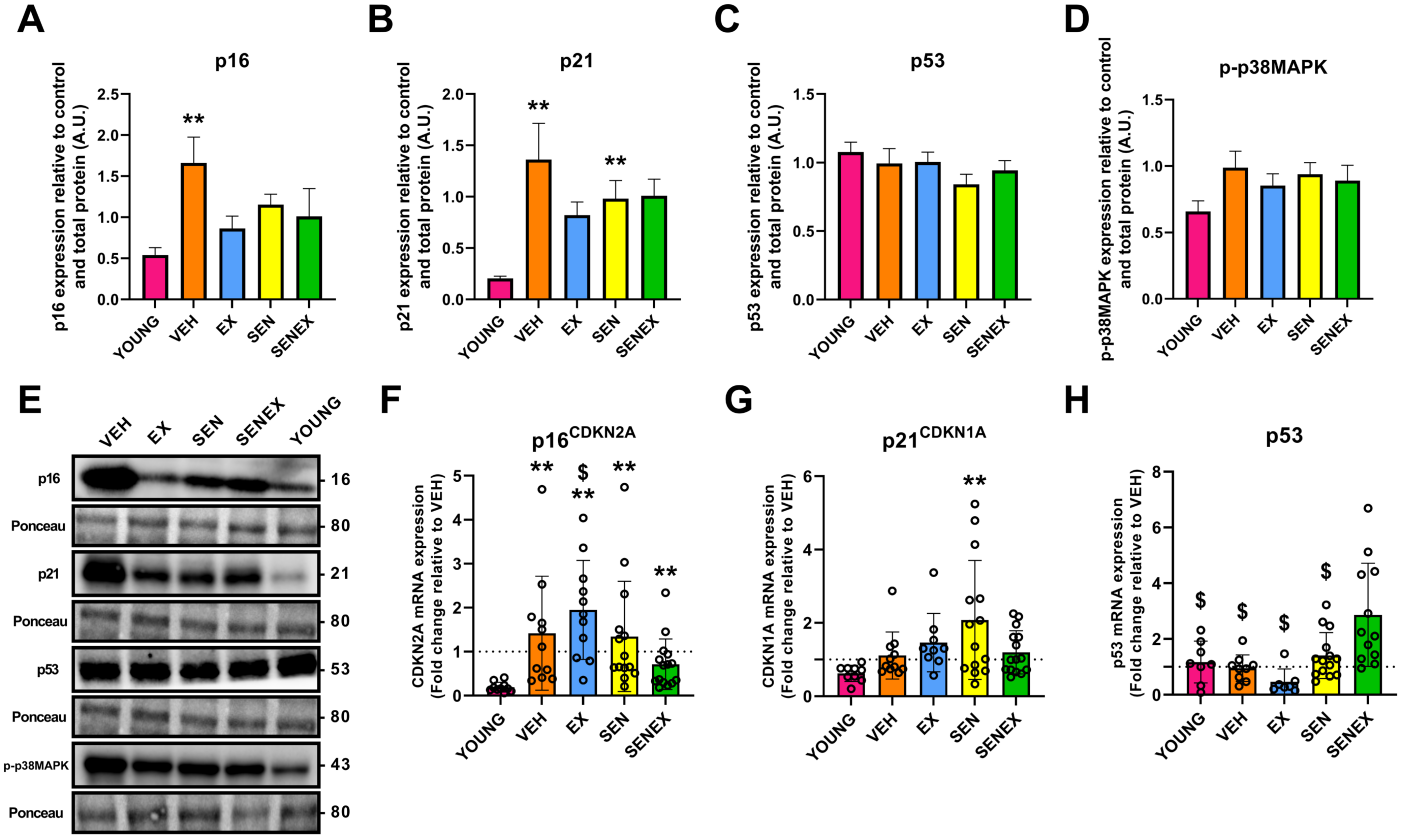
Senescent marker protein expression is higher within the whole heart of aged mice compared to young mice, but gene expression is more variable. (A–D) Protein quantification of senescence markers and inducers within whole heart lysates. (E) Representative bands for each protein and ponceau. Protein levels are normalized to total protein and a between‐gel control sample, expressed in arbitrary units (A.U.). (A) p16 (B) p21 (C) p53 (D) p‐p38MAPK. (F–H) Relative mRNA expression of senescence markers quantified through RT‐qPCR. mRNA values were normalized to reference gene gapdh and expressed relative to the VEH group using the comparative 2^−ΔΔCq^ method. Dashed line represents no fold change (1) and dots represent each mouse. (F) p16^CDKN2A^ (G) p21^CDKN1A^ (H) p53. (A‐H) YOUNG in pink (*n* = 10), VEH in orange (*n* = 12), EX in blue (*n* = 11), SEN in yellow (*n* = 15), and SENEX in green (*n* = 16). All data is presented as mean ± SD. ** denotes value significantly different from YOUNG at *p* < 0.05. $ denotes value significantly different from SENEX at *p* < 0.05.

Gene expression of p16^CDKN2A^ was higher in the VEH (1.42 ± 1.30), EX (1.95 ± 1.13), SEN (1.34 ± 1.25), and SENEX (0.72 ± 0.57), compared to the YOUNG mice (0.19 ± 0.11) (*p* < 0.05) (Figure 3F). For p21^CDKN1A^, only the SEN group (2.08 ± 1.62) displayed significantly higher p21^CDKN1A^ mRNA expression, which was 3.3‐fold higher than YOUNG mice (0.62 ± 0.22) (*p* < 0.05) (Figure 3G), whereas the rest of the interventions showed no difference to the VEH or YOUNG mice (Figure 3G). Interestingly, the SENEX mice displayed the highest p53 mRNA expression (2.86 ± 1.85), which was minimum 2.0‐fold greater than the VEH (0.95 ± 0.48), EX (0.46 ± 0.46), SEN (1.41 ± 0.82), and YOUNG mice (1.17 ± 0.75) (*p* < 0.05) (Figure 3H).

### 2.5 Age or Therapeutic Interventions Do Not Significantly Alter the Gene or Protein Expression of Canonical SASP Components Within the Heart and Circulation

We also assessed whether the expression of canonical SASP factors within the heart was altered, using RT‐qPCR to quantify their relative mRNA expression and western blotting to determine protein expression from whole‐heart lysates. When expressed relative to the VEH group, we found there were no differences in mRNA expression of SASP factors at the whole‐heart level, notably CCL2, CXCL1, CXCL12, NF‐κB, IL‐6, IL‐1α, IL‐β, and TNF‐α (*p* > 0.05) (Figure 4A–H). Similar to gene expression, we found that there were no significant differences in the protein expression of SASP factors TNF‐α, NF‐κB, and TGF‐β across our groups (*p* > 0.05) (Figure 4I–K).

**FIGURE 4:**
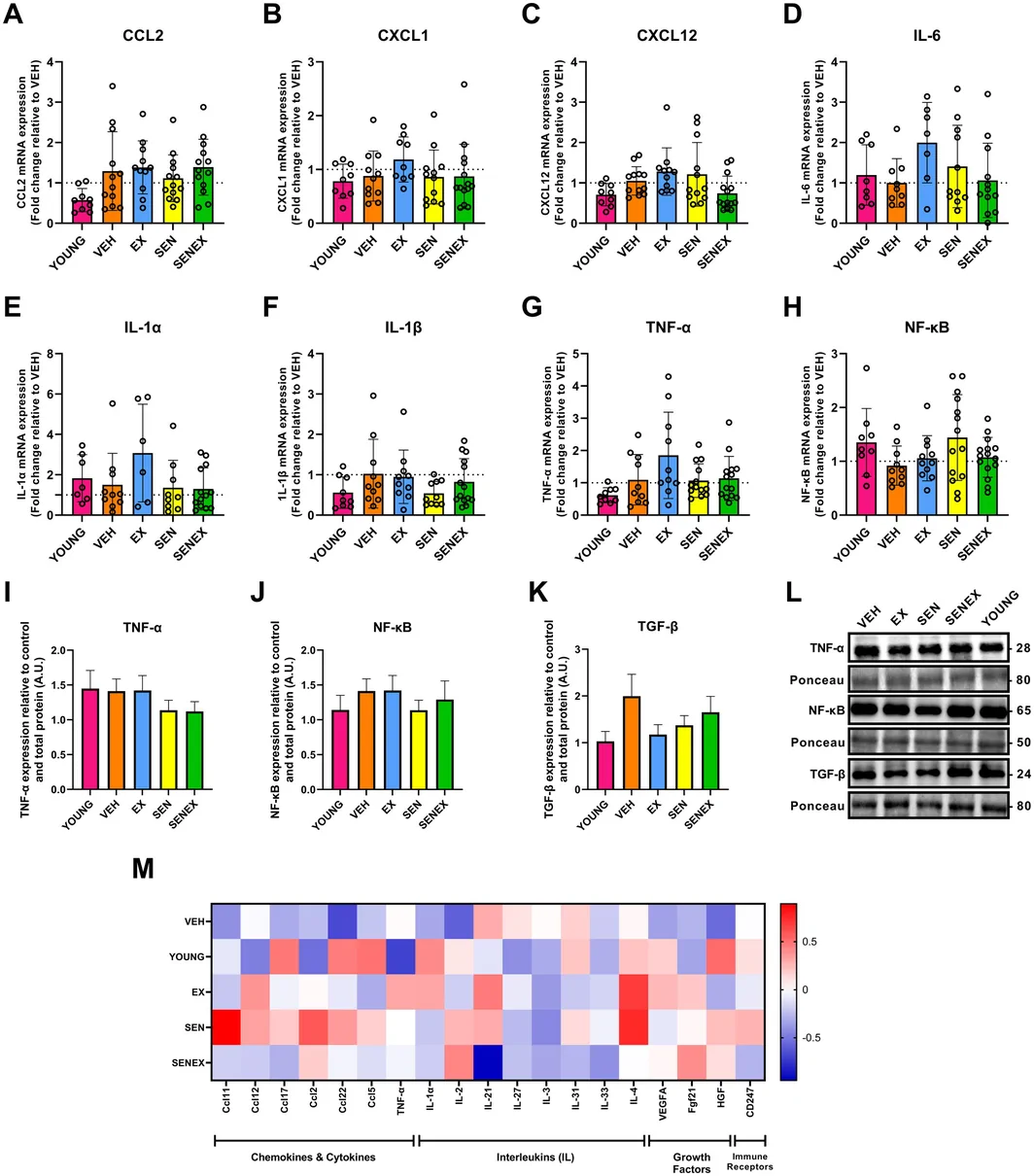
Gene and protein expression of typical SASP components within the whole heart and circulation is unchanged by treatment or age. (A–H) Relative mRNA expression of canonical SASP elements measured using RT‐qPCR. Values were normalized to the arithmetic mean of reference gene gapdh and expressed relative to the VEH group using the comparative 2^−ΔΔCq^ method. Dashed line represents no fold change (1) and dots represent each subject. (A) CCL2 (B) CXCL1 (C) CXCL12 (D) NF‐κB (E) IL‐6 (F) IL‐1α (G) IL‐β (H) TNF‐α. (I–K) Protein quantification of SASP elements within whole heart lysates. (L) Representative bands for each protein and ponceau. Protein levels are normalized to total protein and a between‐gel control sample, expressed in arbitrary units (A.U.). (I) TNF‐α (J) NF‐κB (K) TGF‐β. (A‐K) YOUNG in pink (*n* = 10), VEH in orange (*n* = 12), EX in blue (*n* = 11), SEN in yellow (*n* = 15), and SENEX in green (*n* = 16). All data is presented as mean ± SD. (M) Heatmap of mean *Z*‐scores from each group, showing their relative normalized protein expression (NPX) of SASP factors compared to the overall NPX for each individual protein. *Z*‐scores were calculated per each protein labeled along the *x*‐axis with red = high expression/*Z*‐score and blue = low expression/Z‐score. VEH (*n* = 8), EX (*n* = 8), SEN (*n* = 8), SENEX (*n* = 8), and YOUNG (*n* = 8).

We then employed the use of the Olink target 48 mouse panel using serum to provide a robust quantification of SASP, additional growth factors, and inflammatory signaling molecules or receptors in the circulation (Figure 4M). We found that there were no significant differences across all groups regarding the expression of canonical SASP components within the circulation, including CCL2, CCL12, IL‐1α, TNF‐α, VEGFA, HGF, as well as other inflammatory signaling factors (*p* > 0.05) (Figure 4M).

### 2.6 Expression of Senescence Markers Is Positively Correlated Across Different Cardiac Cell Types and With Worse Cardiac Function

Finally, we reasoned there may be a relationship between senescence markers and cardiac functional measures, so we performed Pearson's correlation analysis between p21+ and p16+ cells and the IVRT, MPI, and relative CO or SV. We found that increases in p21+ and p16+ cells were positively correlated with a higher IVRT and MPI respectively (p21+ & MPI: *p* < 0.001, *r* = 0.50; p21+ & IVRT: *p* < 0.01, *r* = 0.40; p16+ & MPI: *p* < 0.0001, *r* = 0.51; p16+ & IVRT: *p* < 0.0001, *r* = 0.53, Figure 5A–D). Furthermore, increased levels of p21+ and p16+ cells were negatively correlated with relative CO and SV (p21+ & CO: *p* < 0.01, *r* = −0.37; p21+ & SV: *p* < 0.001, *r* = −0.44; p16+ & CO: *p* < 0.01, *r* = −0.37; p16+ & SV: *p* < 0.01, *r* = −0.37, Figure 5E–H). We also assessed senescent marker expression between CMs and ICs and found that p21+ CMs were positively correlated with p16+ ICs (*p* < 0.001, *r* = 0.50, Figure 5I), but p16+ CMs were not correlated with p21+ ICs (*p* > 0.05, *r* = −0.04, Figure 5J). Also, we discovered that p21+ CMs and p21+ ICs were positively correlated (*p* < 0.0001, *r* = 0.91, Figure 5K) and that p16+ CMs and p16+ ICs were positively correlated (*p* < 0.01, *r* = 0.35, Figure 5L).

**FIGURE 5:**
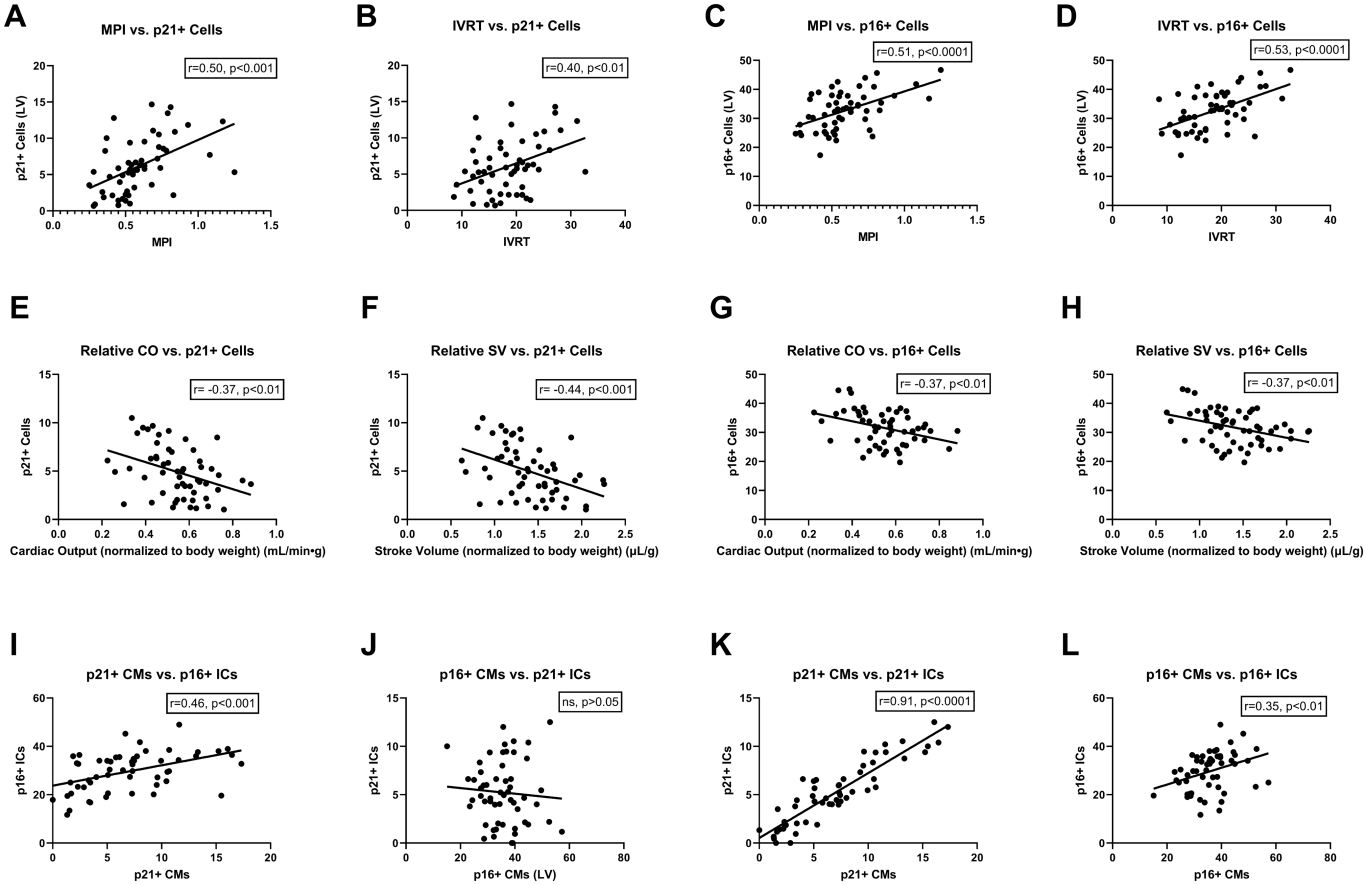
p21+ and p16+ cardiomyocytes and interstitial cells are correlated with each other and with worse cardiac function. (A–L) Pearson correlations between senescence marker expression and cardiac function outcomes. (A) MPI vs. p21+ cells (B) IVRT vs. p21+ cells (C) MPI vs. p16+ cells (D) IVRT vs. p16+ cells (E) Relative CO vs. p21+ cells (F) Relative SV vs. p21+ cells (G) Relative CO vs. p16+ cells (H) Relative SV vs. p16+ cells (I) p21+ CMs vs. p16+ ICs (J) p16+ CMs vs. p21+ ICs (K) p21+ CMs vs. p21+ ICs (L) p16+ CMs vs. p16+ ICs. Values were derived from previous measures from all samples in all groups: VEH (*n* = 10), EX (*n* = 9), SEN (*n* = 13), SENEX (*n* = 14), YOUNG (*n* = 10). Dots represent each sample and their means for each measure on the *x*‐ and *y*‐axis. Pearson's *r* and significance level are provided.

Lastly, we notably included both sexes to provide an inclusive and translatable conclusion, and while beyond the scope of the study, sex‐based analysis was performed and yielded no significant differences for any outcomes (data not shown).

## 3 Discussion

In the present study, we investigated the effects of chronic senolytic administration and aerobic exercise training on the aging of the myocardium and overall heart function. We revealed that 9‐months of D + Q, exercise, or both combined starting at 12‐months of age can lower markers of senescence within CMs and ICs in murine hearts, which was correlated with improved function. However, when these markers were measured at the whole‐heart level, their protein expression was not significantly lowered by the interventions and had inconsistent mRNA expression. This coincided with no significant alteration in the expression of prominent SASP markers within the whole‐heart and circulation. Importantly, we observed no additive effect of combining exercise and senolytics for any of our outcomes. Overall, we highlight an ability of the senolytic D + Q in attenuating myocardial senescence accumulation and present a novel effect of exercise or a combined exercise/D + Q intervention to lower senescence and improve function in the aging heart.

The long‐term administration of senolytics, aerobic exercise training, or both, resulted in a marked decrease in the proportion of p21+, p16+, and y‐H2AX+ CMs and ICs; however, most of these markers were still elevated compared to a young healthy heart. Regardless, the robust clearance of prominent markers suggests a significant decrease in senescence within the aging myocardium. Previous research corroborates this, showing a pronounced ability of D + Q to effectively clear senescent cardiac cells in aged mice (Walaszczyk et al. 2019; Zhu et al. 2015). Importantly, we show for the first time that exercise can reduce senescence markers in the aging heart, building on previous work in the young heart (Werner et al. 2008). As the treatments began prior to and throughout the aging process, our data suggest a protective effect of exercise or D + Q, limiting senescence accumulation. This is an important novel finding, as previous studies have only investigated the acute impact in aged murine hearts when the senescence burden has become significant at old age (~24‐months old).

The accumulation of senescent cells is closely associated with functional deficits in the myocardium, thus examining the effect of our interventions on cardiac function was paramount. Previous work documents that senolytics or exercise improve systolic function (Ehsani et al. 1991; Zhu et al. 2015), however we observed no change in systolic function from age or treatment, using EF or FS. However, the naturally aged VEH mice had a lower relative CO and SV compared to young mice, suggesting reduced systolic efficiency with age. Consistent with this, VEH mice exhibited declines in their EF and FS between 6‐ and 9‐months, an effect partially prevented by senolytic or exercise treatment (Figure S1). This apparent preservation of systolic function was modest, in line with prior work showing limited effects of senolytics on EF (Anderson et al. 2019; Walaszczyk et al. 2019). Collectively, these findings support a growing shift from systolic decline to impaired diastolic function as a dominant feature of cardiac aging contributing to conditions like heart failure with preserved ejection fraction.

The E/A ratio is a frontline indicator of diastolic function, with the Framingham Heart Study showing a clear linear decrease in the E/A ratio with age (Benjamin et al. 1992). After 9‐months, only exercise improved the E/A ratio relative to the VEH, which is consistent with established benefits of aerobic exercise (Subramanian et al. 2025). Aerobic exercise can reduce myocardial stiffness, promoting a stronger and more compliant LV myocardium and thus more efficient early filling of the LV, which may be reflected in the higher E/A ratio (Choi et al. 2009). Interestingly, this effect was not observed after senolytic treatment, either alone or combined with exercise, raising the possibility that chronic D + Q administration does not preserve diastolic function and may attenuate exercise‐induced adaptations. It is possible that early senolytic treatment, prior to substantial senescence accumulation, induces CM cell loss in low‐regenerative tissues such as the myocardium. This reduction in CM number may diminish contractile capacity and promote compensatory hypertrophy of remaining CMs, collectively impairing left ventricular relaxation (Heinzel et al. 2015). This underscores the importance of defining optimal timing and tissues for senolytic interventions to avoid maladaptive outcomes.

Additionally, the E/A ratio is complex and is not a stand‐alone marker of diastolic function. We found that all interventions and the YOUNG group had a significantly lower MPI compared with the VEH, suggestive of better myocardial function. The MPI combines information on systolic (IVCT) and diastolic (IVRT) function—and the MPI, IVCT, and IVRT are known to increase in humans with age (Alhakak et al. 2023). Since all intervention groups also had significantly shorter IVRT compared with VEH mice, the improvement in myocardial performance is more indicative of enhanced diastolic function across all interventions. Therefore, chronic exercise or D + Q treatment may preserve myocardial function during aging, primarily through the maintenance of diastolic function. In fact, from 6‐ to 9‐months there were improvements in IVRT and MPI with each intervention, whereas values remained unchanged in aged VEH mice (Figure S1), indicating functional enhancements. This enhancement may be attributed to the lower senescence burden present, as a high burden likely impairs myocardial function. Consistent with this, senescence marker expression was correlated with a worse MPI, IVRT, CO, and SV. Therefore, by clearing senescent cells, exercise and senolytics can ameliorate these deficits, improving myocardial performance and thus overall cardiac function. Mechanistically, this may occur by attenuating processes that are elevated/impaired with senescence and collectively impair the relaxation or contractility of the myocardium, such as myocardial fibrosis, SERCA mediated calcium handling, and mitochondrial function (Jia et al. 2020; Qin et al. 2013). To our knowledge, we are the first study to explore myocardial function in this context, providing evidence that exercise and/or senolytics can improve myocardial function during aging.

While all our interventions showed beneficial impacts, the combined exercise and senolytic intervention did not produce an additive effect, disagreeing with our hypothesis. Exercise and senolytics independently elicited similar effects, and in some cases restored myocardial functional measures to levels observed in young hearts, yet these effects were not enhanced when the interventions were combined. The absence of any synergistic effect raises important questions regarding the interplay between their individual mechanisms.

The mechanisms underlying the effect of senolytics are well explored and invoke the apoptosis of senescent cells via the disruption of SCAPs, subsequently lowering negative byproducts like SASP, inflammation, ROS, and their burden (Zhu et al. 2015). Conversely, the exact mechanism(s) underlying the impact of aerobic exercise on senescence are poorly understood. Exercise may mitigate potential DNA damage (Werner et al. 2008). Models of accelerated telomere shortening show that short myocardial telomeres repress peroxisome proliferator‐activated receptor‐γ coactivator‐1α (PGC‐1α) (Chang et al. 2016), which may be counteracted by aerobic exercise's potent stimulation of PGC‐1α. In addition, acute immune cell recruitment from exercise was deemed essential to its senolytic effect in skeletal muscle (Jean et al. 2024). Moreover, exercise‐induced adaptations such as mitochondrial biogenesis, improved autophagy, reduced ROS, and lower inflammation have been theorized to repress the senescent burden (Zhang et al. 2022). Altogether, this could limit DDR activation and senescence induction, but further investigation is required to link these underlying changes to senescence repression. This is particularly important since we did not find any change in basal expression of important activators related to the exercise stimulus (PGC‐1α, AMPK, and mTOR) (Figure S3). Regardless, this showcases an exciting prospect for aerobic exercise as an effective strategy to minimize tissue aging and senescence accumulation in the myocardium.

Although the mechanisms underlying the senolytic‐like effect of aerobic exercise are not clear, they likely overlap with senolytics, which may contribute to their lack of additive benefits. For instance, each intervention alone could have hit a ceiling, limiting senescent cell clearance when applied together. Our final assessments were performed at 21‐months of age, when the senescent burden is moderate relative to more advanced ages (Ovadya et al. 2018). Therefore, the available pool of senescent cells may have been insufficient to reveal synergistic effects. This is plausible considering the hit and run nature of senolytic administration, as on the weeks senolytics were not administered, exercise training still occurred. Thus, exercise may clear enough senescent cells during this period that it reduces the available pool, and as D + Q does not remove 100% of senescent cells (Zhu et al. 2015), its efficacy with a limited pool may prevent a meaningful additive effect. From a mechanistic perspective, current evidence suggests a convergence, with both interventions producing shared downstream effects reducing senescence activators like p16, p21, and p53 (Anderson et al. 2019; Lewis‐McDougall et al. 2019; Walaszczyk et al. 2019; Werner et al. 2008) and pro‐inflammatory SASP components (Hickson et al. 2019; Zheng et al. 2019). While our findings do not suggest an additive effect, its potential may depend heavily on senescent burden and intervention timing, highlighting the need for future work to explore treatment timing and mechanistic interactions of combined exercise and senolytic therapies.

We explored a potential underlying mechanism in SASP but did not observe any major increases in SASP factors with age within the heart and circulation or any changes from exercise or senolytics. While this may suggest that chronic exercise or senolytics may not effectively clear SASP factors, the lack of increase in naturally aged mice may indicate that by 21‐months of age the SASP burden may not be as pronounced. In fact, only geriatric mice (> 24‐months‐old) show increases in typical SASP (Ock et al. 2016). Another possibility is the SASP profile of CMs and ICs do not follow a typical phenotype. Recent work showed canonical SASP factors like IL‐6 and TNF‐α did not increase with age, instead, CMs SASP profile contained GDF‐15, EDN3, and TGF‐β2, reflecting more of a hypertrophic and fibrotic influence (Anderson et al. 2019). As CMs have a unique post‐mitotic nature and metabolic demand (Khavinson et al. 2023), it may influence the production of SASP factors. Therefore, using whole heart lysates may make it difficult to disseminate changes in cardiac SASP, representing a limitation in our analysis but highlighting the importance of time and cell type regarding SASPs impact in the myocardium during aging.

We also obtained an overview of protein or gene expression of senescence regulators globally within the heart. Naturally aged hearts exhibited higher p16 and p21 protein expression than young mice, consistent with the age‐related increase in cardiac senescence (Anderson et al. 2019; Lewis‐McDougall et al. 2019). Despite a reduction in these markers in CMs and ICs, neither exercise nor senolytics significantly reduced p16 or p21 mRNA or protein globally. This discrepancy could reflect innate methodological differences, as whole‐heart analyses may obscure cell specific differences that are detectable histologically (Ma'arif et al. 2022). This may be especially prevalent if senescence marker expression is heterogeneous across cardiac cells and the environment, which we show with p21 being expressed at higher levels in the central areas of the LV. Therefore, the location of the whole heart sample may influence the observed protein and gene expression as well. Importantly, both p16 and p21 are subjected to post‐translational regulation (Jung et al. 2010; Ma et al. 2015), which may also help explain the disconnect between the observed mRNA and protein abundance in the VEH. Altogether, these findings suggest that chronic exercise and senolytic treatment may modulate senescence more impactfully at the cellular level with a lesser effect on whole‐heart senescence during early old age.

Similarly, we did not observe an impact of age or treatment on the expression of p‐p38MAPK. This is interesting due to its prominent role in stress‐induced senescence, the more prevalent form within the aging myocardium (Hu et al. 2022). This suggests that the p53/p21 and p16 axes could be the main drivers of senescence in aged hearts, as we report marked increases in p21 and p16 only. However, we did not see this reflected in the protein expression of p53, an upstream activator of p21. There is evidence, in oncogenic cells deficient of p53, that cell cycle arrest and subsequent senescence is initiated by Chk2 mediated upstream pathways that activate p21 independent of p53 (Aliouat‐Denis et al. 2005). Moreover, Chk2 is elevated in response to telomere attrition in aging failing hearts, suggesting a possible role for p53‐independent p21 mediated senescence in the cardiac landscape (Oh et al. 2003). This is consistent with our findings, as we saw no difference in the gene expression of p53 between all groups, minus its elevation in the SENEX hearts, which did not alter protein expression. Overall, it is likely that p‐p38MAPK and p53 still play a role in driving senescence, but it may not be as pronounced in the earlier aging phase, providing novel insight into the dynamics of specific senescent markers during cardiac aging.

This highlights the importance of senescence markers and their heterogeneity across regions and during aging. We found that p21 was a good predictor of both p21 and p16 expression in CMs or ICs, whereas p16 was not. As most senescence markers like p16 and p21 are cell cycle regulators, their activity can also modulate non‐senescent post‐mitotic cells, like CMs, to exit the cell cycle (Ahuja et al. 2007). Young myocardium has been shown to be heterogeneous containing some myocytes that display an aged‐p16 phenotype (Rota et al. 2007), highlighting the importance of being cautious when interpreting and using specific markers to identify senescence, with the best method being a multi‐marker approach that encapsulates different aspects of senescence (i.e., cell cycle arrest, DNA damage and SASP).

In summary, we show for the first time that prolonged senolytic treatment, initiated prior to advanced age, can limit the accumulation of senescent CMs and ICs in the heart. Chronic aerobic exercise produced comparable results, showcasing a novel senolytic‐like effect of exercise in the aging heart. Both aerobic exercise and senolytics markedly improved myocardial function compared to natural aging but had no additive benefits of combining both interventions. Importantly, each intervention could not significantly reduce senescence at the whole heart level, suggesting that the impact of chronic exercise and senolytics may be more pronounced at the cellular level during early old age. This work showcases a promising avenue for attenuating cardiac senescence, positioning both aerobic exercise and senolytics, alone or in combination, as viable strategies to reduce age‐related senescent burden and preserve cardiac function. Further investigation will be required to validate long‐term efficacy, and to define potential systemic effects as these interventions move toward clinical evaluation in human populations.

## 4 Limitations

While this work provides important insight into potential therapeutic strategies to counter cellular senescence in the heart, it is not without its limitations. First, as there is no universally accepted senescence marker, we could not truly identify senescent cells but instead provided a confident assessment of senescence within myocardial cells through the detection of three canonical markers: p16, p21, and γ‐H2AX. Moreover, these markers were assessed in only CMs and ICs, thus lacking insight on how age or exercise/senolytics may influence senescence in important mononuclear cells like fibroblasts, immune cells, or endothelial cells. Second, we did not assess SASP levels or production at the cellular level, providing only a superficial assessment of SASP within the whole‐heart or circulation, which limited our ability to distinguish cell‐specific effects of age or treatment on CMs or other myocardial cell SASP levels. Moreover, we did not assess the impact of age or treatment on mitochondrial dysfunction or oxidative stress. Given the lack of change in SASP observed, we present oxidative stress and mitochondrial dysfunction as key factors to examine in future work regarding exercise and senolytics prevention of senescence in the aging heart. Finally, the endpoint of 21‐month‐old mice may be too early in the aging process to observe significant accumulation of SASP and other aged cardiac phenotypes, limiting our ability to assess the preventative nature of senolytics or exercise on those specific aspects of cardiac aging.

## 5 Materials and Methods

### 5.1 Animals

C57BL/6N mice obtained from Charles River were housed at McMaster University's central animal facility (CAF). Briefly, equal male and female 12‐month‐old mice were randomly assigned to one of four intervention groups (*n* = 20/group): (VEH) naturally aged, (EX) exercise training (30‐min treadmill running; 2×/week), (SEN) senolytic treatment (D + Q, biweekly) or (SENEX) both exercise and senolytics (Figure 1A). All mice completed their assigned intervention for 9‐months, where they reached old age around 21‐months‐old (Figure 1A). A cohort of young healthy mice (*n* = 10) was subjected to the same testing procedures. Following the 9‐month period the sample size and sex breakdown was the following: VEH (7M, 5F), EX (7M, 4F), SEN (7M, 7F), SENEX (9M, 7F), and YOUNG (5M, 5F). Mice were kept in a controlled environment (23°C, 12‐h light/12‐h dark cycle) with unrestricted access to water and chow. All the experiments carried out in this study are documented in the investigator Animal Utilization no. 23–01‐02 approved by McMaster University's animal research ethics board and were conducted in compliance with the guidelines set by the Canadian Council on Animal Care.

Following testing, mice were anesthetized with 2% Isoflurane and blood samples were collected via cardiac puncture. Blood samples clotted for 30 min at room temperature (RT) then spun at 1400 rounds per minute (rpm) for 15 min, with the resulting serum being aliquoted and stored at −80°C. Next, mice were euthanized and the heart was perfused with ice cold 1× phosphate buffered saline (PBS). Hearts were excised and cut transversely with the top half cut longitudinally into two pieces (~30 mg each), both being flash frozen in liquid nitrogen for protein and gene expression analysis. The bottom half was submerged in 15% sucrose in 1X PBS for 1 h then overnight in 30% sucrose in 1X PBS prior to OCT embedding for cryoprotection. OCT embedded ventricles were stored at −80°C until used for immunofluorescent staining.

### 5.2 Senolytic Administration

SEN and SENEX mice were administered a cocktail of D + Q biweekly for 9‐months. Senolytics were given via oral gavage using a 20‐gauge feeding tube with doses based on weight at a ratio of 5 mg/kg for D and 50 mg/kg for Q. The D + Q dose was dissolved in 10% polyethylene glycol 400 (PEG 400) then administered. The dosage and administration technique were based on similar protocols involving D + Q administration in mice (Walaszczyk et al. 2019; Zhu et al. 2015). EX and VEH mice were given the dose of the vehicle (10% PEG 400) via oral gavage biweekly based on weight for the 9‐month intervention.

### 5.3 Treadmill Exercise Training

EX and SENEX mice were subjected to treadmill training on a rodent treadmill apparatus with a modified protocol that has been previously described (Steinberg et al. 2010). Mice completed a 30‐min aerobic exercise training protocol twice a week with a minimum of 2 days rest between sessions. Each session began with a 15‐min warm‐up phase at a speed of 10 m/min, followed by a 15‐min exercise phase at 12.4 m/min with the speed of the exercise phase adjusted weekly to incorporate a progressive training protocol. Mice had access to food and water immediately before and after each session.

### 5.4 High Frequency Ultrasound

HFU analysis was used at 6‐months and 9‐months to obtain a non‐invasive measure of cardiac function using a Prospect T1 high frequency ultrasound (S‐Sharp, Taiwan). Briefly, mice were anesthetized with 2% isoflurane and had the fur covering the upper chest completely removed. Mice were secured to a platform, then had their chest and abdominal region covered with ample ultrasound gel. Short‐axis M‐mode imaging assessed systolic function, including the EDV, ESV, SV, CO, HR, %EF, and %FS. Diastolic function was measured using Pulse‐Wave doppler imaging of the mitral valve inflow from an apical 4‐chamber view, including E wave velocity, A wave velocity, DT, ET, IVRT, and IVCT. After imaging concluded, mice were removed from the platform and anesthetic, followed by recovery in a warming chamber. The images were analyzed using the S‐Sharp Prospect T1 analysis software. Metrics sensitive to mouse size were normalized to body weight: HW, LV Mass, CO, SV, EDV, ESV, and ET. All HFU‐related testing and analysis was performed by blinded investigators.

### 5.5 Immunofluorescence Staining

A comprehensive list of antibodies and their specifications is summarized in Table S1. Briefly, 7‐10 μm cross sections of the ventricles were fixed in 4% PFA for 10‐mins, then washed before being permeabilized in 0.4% Triton X‐100 for 10‐mins. Slides were washed, then blocked for 90‐mins at RT in a solution of 10% GS in 4% BSA in 0.1% Triton X‐100 with the M.O.M. blocking reagent (Vector Laboratories Inc., 30035). Blocked slides were incubated with M.O.M. Protein Concentrate in PBS (1:12.5, Vector Laboratories Inc., 30045) for 5‐mins before incubation with the primary antibody overnight at 4°C, mouse Cardiac Troponin‐T (Abcam, AB8295) at 1:200 in M.O.M. Protein Concentrate in PBS. Slides were washed then incubated with M.O.M. Biotinylated Anti‐Mouse IgG in M.O.M. Protein Concentrate in PBS (1:250, Vector Laboratories Inc., 30,045) for 10‐mins, washed, then incubated in streptavidin, Alexa Fluor 488 conjugate (Invitrogen, S11223) for 60‐mins at RT. Slides were washed, blocked again for 60‐mins at RT, then incubated with the primary antibody overnight at 4°C, rabbit p21 (Abcam, AB109199), p16 (Abcam, AB189034), or γ‐H2AX (Cell Signaling, 9718S) in 1% BSA at 1:200. Slides were washed then incubated with the secondary antibody for 60‐mins at RT, Alexa Fluor 594 goat anti‐rabbit IgG (Invitrogen, A11012), washed, then stained with DAPI 1:20000 for 10‐mins, dried, then mounted with DAKO fluorescent mounting medium (DAKO, S3023). All washes were performed in 1X PBS or PBST, and slides were imaged at 40× magnification using the Nikon Eclipse Ti Microscope and analyzed using Nikon NIS Elements AR 3.2 software (Nikon Instruments, Minato, Tokyo, Japan).

Senescence markers p16, p21, and γ‐H2AX were used to quantify senescent cell populations in the heart using an adapted method from (Redgrave et al. 2023). Positive cells for any marker were identified as having a clean overlap with the nuclear marker DAPI, with CM nuclei being centrally located within cardiac troponin‐T stained fibers and interstitial nuclei located between the fibers. 50 of each cell type were randomly assessed in a 300 × 300 μm square four times each in four different sections of the heart (LV, RV, anterior LV, posterior LV) for a total of 1000 cells per sample. Values were normalized per 100 cells counted.

### 5.6 Protein Extraction and Western Blotting

20–30 mg of the heart was pulverized using a mortar and pestle, with the subsequent powder lysed with ice‐cold radioimmunoprecipitation assay (RIPA) buffer (Sigma‐Aldrich, R0278) containing a Pierce protease inhibitor (Thermo Scientific, 11836153001) and a Roche phosphatase inhibitor (Sigma‐Aldrich, 04906845001). Samples were sonicated and then centrifuged at 14,000*g* for 15 min to pellet debris, from which the purified protein phase was aliquoted out. A bicinchoninic assay (BCA) (Thermo Fisher Scientific, PI23225) determined the protein concentration, and samples were diluted to a final concentration of 2 μg/μL. Total protein (20 μg) was loaded into each lane of a 4%–15% TGX precast gel (Bio‐Rad, 5,678,085) and underwent SDS‐PAGE. Proteins were transferred to a nitrocellulose membrane using the Trans‐Blot Turbo system (Bio‐Rad, 1,704,150). The resulting bands were stained with Ponceau S solution (Sigma‐Aldrich, P7170) to assess equal loading between samples and measure total protein in each lane. Ponceau stained membranes were washed in Tris‐buffered saline with 0.1% Tween‐20 (TBST) then blocked in 5% BSA in TBST for 1 h, then incubated overnight at 4°C with primary antibodies diluted in the block (Table S1). After 16 h, primary antibodies were re‐collected and the membrane was washed with TBST. Next, horseradish peroxidase conjugated secondary antibody (Cell Signaling, Rb: 7074; Ms.: 7076) diluted at 1:10,000 in 5% BSA in TBST was incubated with the membranes for 2 h. Bands were visualized upon the addition of ECL solution (Bio‐Rad, 1,705,062). Images were captured using the ChemiDoc MP Imaging System and quantified on version 6.1.0 Image Lab software (Bio‐Rad, Hercules, California, USA). Bands were normalized to their corresponding Ponceau S image (total protein) plus a between‐gel loading control as samples were compared across multiple gels.

### 5.7 RNA Isolation and Real‐Time Quantitative PCR

Flash frozen heart tissue was removed from −80°C and submerged in Trizol reagent (Invitrogen, 15596018) inside a lysing matrix tube (MP Biomedicals, 116913050). Hearts were lysed using the FastPrep (MP Biomedicals, Irvine, California, USA), and RNA was isolated and purified using the E.Z.N.A Total RNA Kit (Omega Bio‐Tek, R6834‐00). Isolated RNA concentration and its purity were assessed using the Nanodrop 1000 spectrophotometer (Thermo Fisher Scientific, Waltham, Massachusetts, USA), with acceptable samples having 1 μg of total RNA reverse transcribed into cDNA using the high‐capacity cDNA synthesis kit (Thermo Fisher Scientific, 4373966) to a final concentration of 10 ng/μL. cDNA libraries were stored at −20°C until RT‐qPCR was performed. TaqMan assays (Table S2) and Taqman Fast Advanced Master Mix (Thermo Fisher Scientific, 4444557) were used to amplify genes of interest in the QuantStudio 5 real‐time PCR system (Thermo Fisher Scientific, Waltham, Massachusetts, USA). Cq values were obtained in triplicate; values beyond 0.2 cycles of each other in each triplicate were excluded from any analysis. Relative gene expression values were normalized to the mean of reference housekeeping gene gapdh and expressed relative to the VEH group using the comparative 2^−ΔΔCq^ method. A non‐template control group was utilized for each probe to ensure no contamination or non‐specific amplification.

### 5.8 Olink Target 48 Mouse Panel

Serum levels of SASP and pro‐inflammatory factors was assessed using a multiplex immunoassay. The Olink Target 48 Cytokine Panel (Olink Proteomics AB, Uppsala, Sweden) was used, which utilizes Proximity Extension Assay technology to quantify 45 biomarkers with only 1 μL of mouse serum. Briefly, two antibody probes labeled with unique oligonucleotides bind to their respective target proteins and when in close proximity, the oligonucleotides hybridize. DNA polymerase is the added to extend the DNA sequence generating a unique PCR target sequence for each protein. The resulting DNA sequence is detected and quantified using a RT‐PCR instrument (Olink Q100 machine). Samples were run by the Clinical Research Laboratory and Biobank—Genetic and Molecular Epidemiology Laboratory in the Hamilton General Hospital (Hamilton, ON, CAN). Data is provided as normalized protein expression (NPX) as calculated per Olink Target/Flex/Focus Data Analysis manual instructions. For more details on panel validation and the Olink NPX value breakdown can be found on the Olink website (www.olink.com).

### 5.9 Statistical Analysis

One‐way ANOVA was used to determine any statistical significance between interventions at a single timepoint. Two‐way repeated measures ANOVA was used to test for statistically significant differences across multiple timepoints within each intervention group between each timepoint. In the event of a statistically significant ANOVA (*p* < 0.05), multiple comparisons were made using Tukey's post hoc test. Two‐tailed Pearson correlation tests were used to identify relationships between variables. Statistics were conducted using GraphPad Prism 10.3.1 software (GraphPad Software, San Diego, CA, USA).

## Author Contributions

A.L.J. and G.P. designed the experimental outline. R.T.B. and A.L.J. performed all animal experimental procedures. R.T.B. performed all laboratory analysis. B.L.H. and V.A.F. performed all H.F.U. testing and analysis. R.T.B. wrote the manuscript. A.L.J., B.L.H., V.A.F., and G.P. provided edits. G.P., A.L.J., and R.T.B. acquired funding. All authors have read and agreed to the published version of the manuscript.

## Funding

The study was supported by an NSERC discovery grant for G.P. (RGPIN‐2021‐04008), NSERC Canada Graduate Scholarships‐Master's (CGS‐M) program for R.T.B., and CIHR Canada Graduate Scholarships‐PhD (CGS‐D) program for A.L.J.

## Disclosure

Permission Statement: This manuscript is an original work.

## Conflicts of Interest

The authors declare no conflicts of interest.

## Supporting information


**Figure S1:** Chronic exercise, senolytics, or both combined display improvements in systolic and myocardial function. HFU quantification of ejection fraction (EF), fractional shortening (FS), isovolumetric contraction time (IVCT), isovolumetric relaxation time (IVRT), and the myocardial performance index (MPI) from 6‐ to 9‐months. (A) EF, (B) FS, (C) IVCT, (D) IVRT, and (E) MPI. Orange: VEH, blue: EX, yellow: SEN, green: SENEX, pink: YOUNG. Values expressed as mean ± SD. Asterisks denote significant differences: *p < 0.05, **p < 0.01, ***p < 0.001, ****p < 0.0001.
**Figure S2:** Chronic exercise, senolytics, or both combined lower markers of senescence in total cells across different heart regions. Immunofluorescence detection of senescence markers across the whole heart, LV, and RV. (A–C) p21+ cells in the heart (A), LV (B), and the RV (C). (D–F) p16+ cells in the heart (D), LV (E), and the RV (F). (G–I) γ‐H2AX+ cells in the heart (G), LV (H), and the RV (I). Orange: VEH, blue: EX, yellow: SEN, green: SENEX, pink: YOUNG. Values expressed as mean ± SD. Means that do not share a single letter are significantly difference at *p* < 0.05.
**Figure S3:** No differences in baseline expression of major exercise adaptation signaling proteins after 9‐months between interventions. (A) PGC‐1α, (B) AMPK, (C) mTOR. Protein expression from whole heart lysates, normalized to total protein measured through ponceau S staining and a between‐gel control pooled sample. Values expressed as mean ± SEM.
**Table S1:** Immunofluorescence and western blotting primary and secondary antibody Protocols and manufacturer specifications.
**Table S2:** TaqMan probes and reference numbers used for RT‐qPCR.

**Data S1:** Aging cell author checklist. Authors should submit this checklist together with their manuscript. Please ensure that you have read the Author Guidelines in detail before submission.

**Data S2:** Aging cell author checklist. Authors should submit this checklist together with their manuscript. Please ensure that you have read the Author Guidelines in detail before submission.

## Data Availability

Raw data for this study is openly available to verified researchers upon request by contacting the corresponding author.

## References

[acel70759-bib-0001] Ahuja, P. , P. Sdek , and W. R. MacLellan . 2007. “Cardiac Myocyte Cell Cycle Control in Development, Disease, and Regeneration.” Physiological Reviews 87, no. 2: 521–544. 10.1152/physrev.00032.2006. PMC2708177 17429040

[acel70759-bib-0002] Alhakak, A. S. , F. J. Olsen , K. G. Skaarup , et al. 2023. “Changes in Cardiac Time Intervals Over a Decade and the Risk of Incident Heart Failure: The Copenhagen City Heart Study.” International Journal of Cardiology 386: 141–148. 10.1016/j.ijcard.2023.05.018. 37178800

[acel70759-bib-0003] Aliouat‐Denis, C. M. , N. Dendouga , I. Van Den Wyngaert , et al. 2005. “p53‐Independent Regulation of p21Waf1/Cip1 Expression and Senescence by Chk2.” Molecular Cancer Research 3, no. 11: 627–634. 10.1158/1541-7786.MCR-05-0121. 16317088

[acel70759-bib-0004] Anderson, R. , A. Lagnado , D. Maggiorani , et al. 2019. “Length‐Independent Telomere Damage Drives Post‐Mitotic Cardiomyocyte Senescence.” EMBO Journal 38, no. 5: EMBJ2018100492. 10.15252/embj.2018100492. PMC6396144 30737259

[acel70759-bib-0005] Benjamin, E. J. , D. Levy , K. M. Anderson , et al. 1992. “Determinants of Doppler Indexes of Left Ventricular Diastolic Function in Normal Subjects (The Framingham Heart Study).” American Journal of Cardiology 70, no. 4: 508–515. 10.1016/0002-9149(92)91199-E. 1642190

[acel70759-bib-0006] Chang, A. C. Y. , S. G. Ong , E. L. LaGory , et al. 2016. “Telomere Shortening and Metabolic Compromise Underlie Dystrophic Cardiomyopathy.” Proceedings of the National Academy of Sciences of the United States of America 113, no. 46: 13120–13125. 10.1073/pnas.1615340113. PMC5135315 27799523

[acel70759-bib-0007] Chen, M. S. , R. T. Lee , and J. C. Garbern . 2022. “Senescence Mechanisms and Targets in the Heart.” Cardiovascular Research 118, no. 5: 1173–1187. 10.1093/cvr/cvab161. PMC8953446 33963378

[acel70759-bib-0008] Chimenti, C. , J. Kajstura , D. Torella , et al. 2003. “Senescence and Death of Primitive Cells and Myocytes Lead to Premature Cardiac Aging and Heart Failure.” Circulation Research 93, no. 7: 604–613. 10.1161/01.RES.0000093985.76901.AF. 12958145

[acel70759-bib-0009] Choi, S. Y. , H. J. Chang , S. I. Choi , et al. 2009. “Long‐Term Exercise Training Attenuates Age‐Related Diastolic Dysfunction: Association of Myocardial Collagen Cross‐Linking.” Journal of Korean Medical Science 24, no. 1: 32. 10.3346/jkms.2009.24.1.32. PMC2650965 19270810

[acel70759-bib-0010] Coppé, J. P. , P. Y. Desprez , A. Krtolica , and J. Campisi . 2010. “The Senescence‐Associated Secretory Phenotype: The Dark Side of Tumor Suppression.” Annual Review of Pathology: Mechanisms of Disease 5: 99–118. 10.1146/annurev-pathol-121808-102144. PMC4166495 20078217

[acel70759-bib-0011] Ehsani, A. A. , T. Ogawa , T. R. Miller , R. J. Spina , and S. M. Jilka . 1991. “Exercise Training Improves Left Ventricular Systolic Function in Older Men.” Circulation 83, no. 1: 96–103. 10.1161/01.cir.83.1.96. 1984902

[acel70759-bib-0012] Hayflick, L. , and P. S. Moorhead . 1961. “The Serial Cultivation of Human Diploid Cell Strains.” Experimental Cell Research 25, no. 3: 585–621. 10.1016/0014-4827(61)90192-6. 13905658

[acel70759-bib-0013] Heinzel, F. R. , F. Hohendanner , G. Jin , S. Sedej , and F. Edelmann . 2015. “Myocardial Hypertrophy and Its Role in Heart Failure With Preserved Ejection Fraction.” Journal of Applied Physiology 119, no. 10: 1233–1242. 10.1152/japplphysiol.00374.2015. PMC4652334 26183480

[acel70759-bib-0014] Hernandez‐Segura, A. , J. Nehme , and M. Demaria . 2018. “Hallmarks of Cellular Senescence.” Trends in Cell Biology 28, no. 6: 436–453. 10.1016/j.tcb.2018.02.001. 29477613

[acel70759-bib-0015] Herranz, N. , and J. Gil . 2018. “Mechanisms and Functions of Cellular Senescence.” Journal of Clinical Investigation 128, no. 4: 1238–1246. 10.1172/JCI95148. PMC5873888 29608137

[acel70759-bib-0016] Hickson, L. T. J. , L. G. P. Langhi Prata , S. A. Bobart , et al. 2019. “Senolytics Decrease Senescent Cells in Humans: Preliminary Report From a Clinical Trial of Dasatinib Plus Quercetin in Individuals With Diabetic Kidney Disease.” eBioMedicine 47: 446–456. 10.1016/j.ebiom.2019.08.069. PMC6796530 31542391

[acel70759-bib-0017] Hoare, M. , Y. Ito , T. W. Kang , et al. 2016. “NOTCH1 Mediates a Switch Between Two Distinct Secretomes During Senescence.” Nature Cell Biology 18, no. 9: 979–992. 10.1038/ncb3397. PMC5008465 27525720

[acel70759-bib-0018] Hu, C. , X. Zhang , T. Teng , Z. G. Ma , and Q. Z. Tang . 2022. “Cellular Senescence in Cardiovascular Diseases: A Systematic Review.” Aging and Disease 13, no. 1: 103. 10.14336/AD.2021.0927. PMC8782554 35111365

[acel70759-bib-0019] Jean, W. H. , Y. C. Lin , P. Y. Ang , et al. 2024. “Senolytic Effects of Exercise in Human Muscles Require Acute Inflammation.” Aging 16, no. 10: 8599–8610. 10.18632/aging.205827. PMC11164480 38752873

[acel70759-bib-0020] Jia, K. , Y. Dai , A. Liu , et al. 2020. “Senolytic Agent Navitoclax Inhibits Angiotensin II‐Induced Heart Failure in Mice.” Journal of Cardiovascular Pharmacology 76, no. 4: 452–460. 10.1097/FJC.0000000000000878. 32675749

[acel70759-bib-0021] Jung, Y. S. , Y. Qian , and X. Chen . 2010. “Examination of the Expanding Pathways for the Regulation of p21 Expression and Activity.” Cellular Signalling 22, no. 7: 1003–1012. 10.1016/j.cellsig.2010.01.013. PMC2860671 20100570

[acel70759-bib-0022] Khavinson, V. , N. Linkova , A. Dyatlova , R. Kantemirova , and K. Kozlov . 2023. “Senescence‐Associated Secretory Phenotype of Cardiovascular System Cells and Inflammaging: Perspectives of Peptide Regulation.” Cells 12, no. 1: 106. 10.3390/cells12010106. PMC9818427 36611900

[acel70759-bib-0023] Lewis‐McDougall, F. C. , P. J. Ruchaya , E. Domenjo‐Vila , et al. 2019. “Aged‐Senescent Cells Contribute to Impaired Heart Regeneration.” Aging Cell 18, no. 3: e12931. 10.1111/acel.12931. PMC6516154 30854802

[acel70759-bib-0024] Lim, C. C. , C. S. Apstein , W. S. Colucci , and R. Liao . 2000. “Impaired Cell Shortening and Relengthening With Increased Pacing Frequency Are Intrinsic to the Senescent Mouse Cardiomyocyte.” Journal of Molecular and Cellular Cardiology 32, no. 11: 2075–2082. 10.1006/jmcc.2000.1239. 11040110

[acel70759-bib-0025] Luan, Y. , X. Zhu , Y. Jiao , et al. 2024. “Cardiac Cell Senescence: Molecular Mechanisms, Key Proteins and Therapeutic Targets.” Cell Death Discovery 10, no. 1: 78. 10.1038/s41420-023-01792-5. PMC10866973 38355681

[acel70759-bib-0026] Ma, W. L. , L. Wang , L. X. Liu , and X. L. Wang . 2015. “Effect of Phosphorylation and Methylation on the Function of the p16INK4a Protein in Non‐Small Cell Lung Cancer A549 Cells.” Oncology Letters 10, no. 4: 2277–2282. 10.3892/ol.2015.3617. PMC4579901 26622834

[acel70759-bib-0027] Ma'arif, B. , F. A. Sholihah , A. Mahardiani , B. Fauziyah , D. M. Mirza , and M. Agil . 2022. “Runt‐Related Transcription Factor 2 (Runx2) Measurement in Phytoestrogen‐Induced Bone: A Comparison of Western Blot and Immunohistochemistry Methods.” Biomedical and Pharmacology Journal 15, no. 2: 1039–1052. 10.13005/bpj/2439.

[acel70759-bib-0028] Martini, H. , L. Lefevre , S. Sayir , et al. 2021. “Selective Cardiomyocyte Oxidative Stress Leads to Bystander Senescence of Cardiac Stromal Cells.” International Journal of Molecular Sciences 22, no. 5: 2245. 10.3390/ijms22052245. PMC7956294 33668142

[acel70759-bib-0029] Ock, S. , W. S. Lee , J. Ahn , et al. 2016. “Deletion of IGF‐1 Receptors in Cardiomyocytes Attenuates Cardiac Aging in Male Mice.” Endocrinology 157, no. 1: 336–345. 10.1210/en.2015-1709. PMC4701888 26469138

[acel70759-bib-0030] Oh, H. , S. C. Wang , A. Prahash , et al. 2003. “Telomere Attrition and Chk2 Activation in Human Heart Failure.” Proceedings of the National Academy of Sciences of the United States of America 100, no. 9: 5378–5383. 10.1073/pnas.0836098100. PMC154353 12702777

[acel70759-bib-0031] Ovadya, Y. , T. Landsberger , H. Leins , et al. 2018. “Impaired Immune Surveillance Accelerates Accumulation of Senescent Cells and Aging.” Nature Communications 9, no. 1: 5435. 10.1038/s41467-018-07825-3. PMC6303397 30575733

[acel70759-bib-0032] Qin, F. , D. A. Siwik , S. Lancel , et al. 2013. “Hydrogen Peroxide‐Mediated SERCA Cysteine 674 Oxidation Contributes to Impaired Cardiac Myocyte Relaxation in Senescent Mouse Heart.” Journal of the American Heart Association 2, no. 4: e000184. 10.1161/JAHA.113.000184. PMC3828801 23963753

[acel70759-bib-0033] Qiu, Y. , B. Fernández‐García , H. I. Lehmann , et al. 2025. “Exercise Attenuates the Hallmarks of Aging: Novel Perspectives.” Journal of Sport and Health Science 15: 101108. 10.1016/j.jshs.2025.101108. PMC13158393 41352451

[acel70759-bib-0034] Redgrave, R. E. , E. Dookun , L. K. Booth , et al. 2023. “Senescent Cardiomyocytes Contribute to Cardiac Dysfunction Following Myocardial Infarction.” NPJ Aging 9, no. 1: 15. 10.1038/s41514-023-00113-5. PMC10267185 37316516

[acel70759-bib-0035] Ribeiro, A. S. F. , B. E. Zerolo , F. López‐Espuela , R. Sánchez , and V. S. Fernandes . 2023. “Cardiac System During the Aging Process.” Aging and Disease 14, no. 4: 1105. 10.14336/AD.2023.0115. PMC10389818 37163425

[acel70759-bib-0036] Robles, S. J. , and G. R. Adami . 1998. “Agents That Cause DNA Double Strand Breaks Lead to p16(INK4a) Enrichment and the Premature Senescence of Normal Fibroblasts.” Oncogene 16, no. 9: 1113–1123. 10.1038/sj.onc.1201862. 9528853

[acel70759-bib-0037] Rota, M. , T. Hosoda , A. De Angelis , et al. 2007. “The Young Mouse Heart Is Composed of Myocytes Heterogeneous in Age and Function.” Circulation Research 101, no. 4: 387–399. 10.1161/CIRCRESAHA.107.151449. 17601802

[acel70759-bib-0038] Steinberg, G. R. , H. M. O'Neill , N. L. Dzamko , et al. 2010. “Whole Body Deletion of AMP‐Activated Protein Kinase β2 Reduces Muscle AMPK Activity and Exercise Capacity.” Journal of Biological Chemistry 285, no. 48: 37198–37209. 10.1074/jbc.M110.102434. PMC2988326 20855892

[acel70759-bib-0039] Subramanian, V. , W. J. Tucker , A. E. Peters , B. Upadhya , D. W. Kitzman , and A. Pandey . 2025. “Cardiovascular Aging and Exercise: Implications for Heart Failure Prevention and Management.” Circulation Research 137, no. 2: 205–230. 10.1161/CIRCRESAHA.125.325531. 40608862

[acel70759-bib-0040] Walaszczyk, A. , E. Dookun , R. Redgrave , et al. 2019. “Pharmacological Clearance of Senescent Cells Improves Survival and Recovery in Aged Mice Following Acute Myocardial Infarction.” Aging Cell 18, no. 3: e12945. 10.1111/acel.12945. PMC6516151 30920115

[acel70759-bib-0041] Wang, E. 1995. “Senescent Human Fibroblasts Resist Programmed Cell Death, and Failure to Suppress bcl2 Is Involved.” Cancer Research 5585, no. 112: 2284–2292. 7757977

[acel70759-bib-0042] Werner, C. , M. Hanhoun , T. Widmann , et al. 2008. “Effects of Physical Exercise on Myocardial Telomere‐Regulating Proteins, Survival Pathways, and Apoptosis.” Journal of the American College of Cardiology 52, no. 6: 470–482. 10.1016/j.jacc.2008.04.034. 18672169

[acel70759-bib-0043] Zhang, X. , D. A. Englund , Z. Aversa , S. K. Jachim , T. A. White , and N. K. Lebrasseur . 2022. “Exercise Counters the Age‐Related Accumulation of Senescent Cells.” Exercise and Sport Sciences Reviews 50, no. 4: 213–221. 10.1249/JES.0000000000000302. PMC9680689 35776782

[acel70759-bib-0044] Zheng, G. , P. Qiu , R. Xia , et al. 2019. “Effect of Aerobic Exercise on Inflammatory Markers in Healthy Middle‐Aged and Older Adults: A Systematic Review and Meta‐Analysis of Randomized Controlled Trials.” Frontiers in Aging Neuroscience 11: 98. 10.3389/fnagi.2019.00098. PMC6497785 31080412

[acel70759-bib-0045] Zhu, Y. , T. Tchkonia , T. Pirtskhalava , et al. 2015. “The Achilles' Heel of Senescent Cells: From Transcriptome to Senolytic Drugs.” Aging Cell 14, no. 4: 644–658. 10.1111/acel.12344. PMC4531078 25754370

