# Supplementary material for: Chronic Senolytic Treatment and/or Aerobic Exercise Reduce Senescence and Improve Myocardial Function During Aging in the Heart
Source: Aging Cell. 2026 Oct 7;25(10):e70759. doi: 10.1111/acel.70759 (PMC13644388; doi:10.1111/acel.70759)
Supplement: Supplementary file 1 [file ACEL-25-e70759-s001.pdf]

AGING CELL AUTHOR CHECKLIST. ***Authors should submit this checklist together with their manuscript. Please ensure that you have read the Author Guidelines in detail before submission.***

**Title**	Chronic senolytic treatment and/or aerobic exercise reduce senescence and improve myocardial function during aging in the heart						
**Authors**	Ryan T. Bevington, Amanda L. Johnson, Briana L. Hockey, Val A. Fajardo, Gianni Parise						
**Manuscript Type**	Original Research Article						
**Total Character Count (including spaces)[1]**	49, 976						
**Word count of Summary[2]**	250 words						
**Number of papers cited in the References[3]**	45						
**Listing of all Tables (Table1, Table 2 etc)[4]**	Table 1, Table 2						
#1	Table 1. Anthropomorphic Measurements.						
#2	Table 2. General cardiac function and LV filling metrics largely unaffected by age or treatment.						
**Figure specifications (please complete one row per figure)[5]**<br><br>***Figure no.***	Colour<br>***(yes/no)***	Greyscale<br>***(yes/no)***	Black and white<br>***(yes/no)***	Single column (80mm)<br>***(yes/no)***	Double column (180mm)<br>***(yes/no)***	Size of figure at full scale (mm x mm)<br>***(insert details)***	Smallest font size used in the figure at full scale (minimum 6pt)<br>***(insert details)***
#1	Yes	No	No	No	Yes	209.6 x 182.5	8pt
#2	Yes	No	No	No	Yes	214.1 x 278.9	11pt
#3	Yes	No	No	No	Yes	215.2 x 147.2	6pt
#4	Yes	No	No	No	Yes	209.9 x 269.2	7pt
#5	Yes	No	No	No	Yes	259.2 x 113.4	10pt

[1] The maximum character count allowed is 50,000 (incl. spaces) for Primary Research Papers and Reviews, 10,000 for Short Takes.

[2] Summary should not exceed 250 words.

[3] Primary Research Papers can contain a maximum of two tables. If more are needed they should replace some of the Figures or can be placed in the Supporting Information.

[4] A maximum of 45 references is allowed for Primary Research Papers and 20 references for Short Takes.

[5] A Primary Research Paper may contain up to 6 figures and a Short Take up to 2 figures. Authors are encouraged to provide figures in the size they are to appear in the journal and at the specifications given.
