# Supplementary material for: Chronic Senolytic Treatment and/or Aerobic Exercise Reduce Senescence and Improve Myocardial Function During Aging in the Heart
Source: Aging Cell. 2026 Oct 7;25(10):e70759. doi: 10.1111/acel.70759 (PMC13644388; doi:10.1111/acel.70759)
Supplement: Supplementary file 3 [file ACEL-25-e70759-s003.docx]

**Supporting Information File**

**Supplementary Table 1.** Immunofluorescence and Western Blotting Primary and Secondary Antibody Protocols and Manufacturer Specifications.

|  | **Primary Antibody** | **Application** | **Species** | **Dilution** | **Block** | **Secondary Antibody** | **Manufacturer** |
| --- | --- | --- | --- | --- | --- | --- | --- |
| **1** | Cardiac troponin-T | IF | Mouse | 1:200 | 10% goat serum in 4% BSA in 0.1 Triton X-100 in PBS w/ M.O.M.® blocking reagent | Streptavidin, Alexa Fluor™ 488, Conjugate (Invitrogen, S11223) | Abcam (ab8295) |
| **2** | p16 | IF | Rabbit | 1:100 | 10% goat serum in 4% BSA in 0.1 Triton X-100 in PBS | Alexa Fluor™ 594 (Invitrogen, A11012) | Abcam (ab189034) |
| **3** | p21 | IF | Rabbit | 1:100 | 10% goat serum in 4% BSA in 0.1 Triton X-100 in PBS | Alexa Fluor™ 594 (Invitrogen, A11012) | Abcam (ab109199) |
| **4** | γ-H2AX | IF | Rabbit | 1:200 | 10% goat serum in 4% BSA in 0.1 Triton X-100 in PBS | Alexa Fluor™ 594 (Invitrogen, A11012) | Cell Signaling (9718S) |
| **5** | p16 | WB | Rabbit | 1:1000 | 5% BSA in TBST | Anti-rabbit IgG, HRP-linked Antibody (Cell Signaling, 7074S) | Abcam (ab189034) |
| **6** | p21 | WB | Mouse | 1:1000 | 5% BSA in TBST | Anti-mouse IgG, HRP-linked Antibody (Cell Signaling, 7076S) | Cell Signaling (DCS60) |
| **7** | p53 | WB | Rabbit | 1:1000 | 5% BSA in TBST | Anti-rabbit IgG, HRP-linked Antibody (Cell Signaling, 7074S) | Cell Signaling (9282S) |
| **8** | p-p38MAPK | WB | Rabbit | 1:1000 | 5% BSA in TBST | Anti-rabbit IgG, HRP-linked Antibody (Cell Signaling, 7074S) | Cell Signaling (3D7) |
| **9** | TNF-α | WB | Rabbit | 1:1000 | 5% BSA in TBST | Anti-rabbit IgG, HRP-linked Antibody (Cell Signaling, 7074S) | Cell Signaling (D2D4) |
| **10** | NF-κB | WB | Rabbit | 1:1000 | 5% BSA in TBST | Anti-rabbit IgG, HRP-linked Antibody (Cell Signaling, 7074S) | Cell Signaling (D14E12) |
| **11** | TGF-β | WB | Rabbit | 1:1000 | 5% BSA in TBST | Anti-rabbit IgG, HRP-linked Antibody (Cell Signaling, 7074S) | Cell Signaling (3711S) |
| **12** | PGC-1α | WB | Rabbit | 1:1000 | 5% BSA in TBST | Anti-rabbit IgG, HRP-linked Antibody (Cell Signaling, 7074S) | Cell Signaling (3G6) |
| **13** | AMPK | WB | Rabbit | 1:1000 | 5% BSA in TBST | Anti-rabbit IgG, HRP-linked Antibody (Cell Signaling, 7074S) | Cell Signaling (25325) |
| **14** | mTOR | WB | Rabbit | 1:1000 | 5% BSA in TBST | Anti-rabbit IgG, HRP-linked Antibody (Cell Signaling, 7074S) | Cell Signaling (7C10) |

**Supplementary Table 2.** TaqMan Probes and Reference Numbers used for RT-qPCR

|  | **Gene** | **Assay ID** | **Marker** |
| --- | --- | --- | --- |
| **1** | Cdkn1a (p21) | Mm00432448_m1 | Senescence |
| **2** | Cdkn2a (p16) | Mm01257348_m1 | Senescence |
| **3** | Ccl2 | Mm00441242_m1 | Inflammation (SASP) |
| **4** | Cxcl1 | Mm04207460_m1 | Inflammation (SASP) |
| **5** | Cxcl12 | Mm00445553_m1 | Inflammation (SASP) |
| **6** | Gapdh | Mm99999915_g1 | Housekeeping gene |
| **7** | Il-1α | Mm00439620_m1 | Inflammation (SASP) |
| **8** | Il-1β | Mm00434228_m1 | Inflammation (SASP) |
| **9** | Il-6 | Mm00446190_m1 | Inflammation (SASP) |
| **10** | Nfkb1 | Mm00476361_m1 | Inflammation (SASP) |
| **11** | Tnf-α | Mm00443258_m1 | Inflammation (SASP) |
| **12** | Trp53 | Mm01731287_m1 | Senescence |

**Supplementary Figure 1. Chronic exercise, Senolytics, or both combined display improvements in systolic and myocardial function.** HFU quantification of ejection fraction (EF), fractional shortening (FS), isovolumetric contraction time (IVCT), isovolumetric relaxation time (IVRT), and the myocardial performance index (MPI) from 6- to 9-months. (A) EF, (B) FS, (C) IVCT, (D) IVRT, and (E) MPI. Orange: VEH, blue: EX, yellow: SEN, green: SENEX, pink: YOUNG. Values expressed as mean ± SD. Asterisks denote significant differences: * p<0.05, ** p<0.01, *** p<0.001, **** p<0.0001.

**Supplementary Figure 2. Chronic exercise, Senolytics, or both combined lower markers of senescence in total cells across different heart regions.** Immunofluorescence detection of senescence markers across the whole heart, LV, and RV. (A-C) p21+ cells in the heart (A), LV (B), and the RV (C). (D-F) p16+ cells in the heart (D), LV (E), and the RV (F). (G-I) γ-H2AX+ cells in the heart (G), LV (H), and the RV (I). Orange: VEH, blue: EX, yellow: SEN, green: SENEX, pink: YOUNG. Values expressed as mean ± SD. Means that do not share a single letter are significantly difference at p<0.05.

**Supplementary Figure 3. No differences in baseline expression of major exercise adaptation signalling proteins after 9-months between interventions.** (A) PGC-1α, (B) AMPK, (C) mTOR. Protein expression from whole heart lysates, normalized to total protein measured through ponceau S staining and a between-gel control pooled sample. Values expressed as mean ± SEM.
